# From biomarker to bedside? A critical commentary on ischemia-modified albumin in preeclampsia

**DOI:** 10.61622/rbgo/2025rbgo97

**Published:** 2025-12-09

**Authors:** Seshadri Reddy Varikasuvu

**Affiliations:** 1 All India Institute of Medical Sciences Department of Biochemistry Deoghar India Department of Biochemistry, All India Institute of Medical Sciences, Deoghar, India.

Dear Editor,

We read with interest the article by Zuhal et al.,^(1)^ which compared serum ischemia-modified albumin (IMA) levels between preeclamptic and healthy pregnant women. The authors deserve credit for addressing a potential biomarker that may elucidate oxidative stress mechanisms in preeclampsia (PE). The observed increase in IMA levels among preeclamptic patients is consistent with established knowledge of oxidative imbalance in PE. However, the diagnostic performance reported (AUC: 0.690) was only moderate, and the lack of serum albumin measurements precluded calculation of the IMA/albumin ratio (IMAR), which prior studies suggest could improve diagnostic precision.^(2,3)^

Several investigations have demonstrated progressive rises in IMA from normal pregnancies to more severe forms of PE.^(4-10)^ Elevated IMA has also been associated with hypertension, deranged hepatic enzymes, and adverse neonatal outcomes such as fetal growth restriction (FGR).^(5,6)^ Teselkin et al. reported correlations between IMA and antioxidant status, reinforcing the oxidative stress link in PE.^(4)^ Similarly, higher serum IMA levels were documented in preeclamptic women versus controls.^(7-9)^ Further, D’Souza et al.^(3)^ provided early evidence for salivary IMA and IMAR as useful in identifying PE severity. Notably, salivary IMAR distinguished controls from mild PE and correlated inversely with birthweight, raising the possibility of future non-invasive screening. Mokhtar et al.^(5)^ showed significantly higher IMA levels in severe PE compared to mild cases and reported strong diagnostic accuracy (sensitivity 82.5%, specificity 77.5%, AUC 0.889). Conversely, Karaşin et al. ^(11)^ and Zuhal et al.^(1)^ did not find significant differences between mild and severe PE, though both confirmed higher IMA in PE than in normotensive pregnancies. Karaşin et al. reported significant differences (p = 0.003 and 0.001) between PE groups and controls, but not between mild and severe PE (p = 0.191).^(11)^

Beyond diagnostic comparisons, other studies have highlighted IMA's broader clinical implications. Parasher et al.^(12)^ demonstrated elevations of both IMA and neutrophil gelatinase–associated lipocalin (NGAL) in PE, implicating renal stress and endothelial dysfunction. Gupta et al.^(13)^ reported positive correlations of IMA with creatinine, uric acid, and proteinuria, and negative correlations with bilirubin and albumin, supporting its role as an integrative marker of systemic stress. Jaiswar et al.^(6)^ linked increased IMA with placental pathology and poor neonatal outcomes. Schoots et al.^(7)^ found higher IMA in PE-related FGR compared to isolated FGR and controls, along with an inverse association with free thiols, suggesting IMA may differentiate oxidative pathways in FGR subtypes. Complementing maternal data, Ozmert et al.^(10)^ reported elevated cord blood IMA in preterm infants of preeclamptic mothers, associated with inflammation and increased risk of small-for-gestational-age births.

Together, these findings underscore IMA's potential as a clinically relevant biomarker in PE. Nevertheless, variability in assay methods, study designs, and patient populations complicates interpretation. While most studies confirm elevated IMA in PE, discrepancies remain regarding its capacity to stratify disease severity. Correcting IMA for albumin (IMAR) appears promising, especially given links to fetal outcomes and its feasibility in saliva or cord blood. Future research should: (i) routinely assess IMAR alongside IMA; (ii) stratify findings by mild versus severe PE; (iii) investigate associations with maternal, fetal, and neonatal outcomes; (iv) incorporate longitudinal sampling across gestation; and (v) explore salivary and cord blood IMAR as non-invasive maternal and neonatal markers.^(3,10)^

To conclude, IMA is a promising yet complex biomarker of PE. Consistent elevations across studies highlight its link to oxidative stress, but its diagnostic accuracy varies. Incorporating IMAR, integrating IMA with angiogenic and inflammatory markers, and focusing on longitudinal outcome-linked analyses may enhance its value. Establishing standardized methodologies and robust clinical validation will determine whether IMA can be reliably implemented in obstetric practice.
